# The genome sequence of *Dyella jiangningensis* FCAV SCS01 from a lignocellulose-decomposing microbial consortium metagenome reveals potential for biotechnological applications

**DOI:** 10.1590/1678-4685-GMB-2017-0155

**Published:** 2018-05-14

**Authors:** Joana G. Desiderato, Danillo O. Alvarenga, Milena T.L. Constancio, Lucia M.C. Alves, Alessandro M. Varani

**Affiliations:** 1 Universidade Estadual Paulista Universidade Estadual Paulista Faculdade de Ciências Agrárias e Veterinárias Departamento de Tecnologia JaboticabalSP Brazil Departamento de Tecnologia, Faculdade de Ciências Agrárias e Veterinárias, Universidade Estadual “Júlio de Mesquita Filho” (UNESP), Jaboticabal, SP, Brazil

**Keywords:** Plant cell wall polysaccharides, glycoside hydrolases, genome annotation, comparative genomics, Rhodanobacteraceae

## Abstract

Cellulose and its associated polymers are structural components of the plant cell wall, constituting one of the major sources of carbon and energy in nature. The carbon cycle is dependent on cellulose- and lignin-decomposing microbial communities and their enzymatic systems acting as consortia. These microbial consortia are under constant exploration for their potential biotechnological use. Herein, we describe the characterization of the genome of *Dyella jiangningensis* FCAV SCS01, recovered from the metagenome of a lignocellulose-degrading microbial consortium, which was isolated from a sugarcane crop soil under mechanical harvesting and covered by decomposing straw. The 4.7 Mbp genome encodes 4,194 proteins, including 36 glycoside hydrolases (GH), supporting the hypothesis that this bacterium may contribute to lignocellulose decomposition. Comparative analysis among fully sequenced *Dyella* species indicate that the genome synteny is not conserved, and that *D. jiangningensis* FCAV SCS01 carries 372 unique genes, including an alpha-glucosidase and maltodextrin glucosidase coding genes, and other potential biomass degradation related genes. Additional genomic features, such as prophage-like, genomic islands and putative new biosynthetic clusters were also uncovered. Overall, *D. jiangningensis* FCAV SCS01 represents the first South American *Dyella* genome sequenced and shows an exclusive feature among its genus, related to biomass degradation.

Plant cell wall structural molecules are primarily represented by cellulose and its associated polymers, such as hemicellulose and lignin. These organic components are very stable and robust, protecting plant cell contents from the environment. Additionally, these polysaccharides are important sources of carbon and energy which are constantly accumulated and released into the environment during plant development or human manipulation by agriculture. While on an industrial scale the cellulosic material is commonly used for the development of wood, paper and others derivatives, in nature it is assimilated and cycled through cellulose- and lignin-degrading microbial communities, which are the main keepers of carbon and energy cycles on a global scale (Bayer *et al.*, 2013). These communities are commonly referred to as microbial consortia, due to their abilities to work in cooperation to degrade and or metabolize compounds.

Although interactions between microbial consortia and the environment have been occurring for billions of years, only during the past decades have the lignocellulose degrading enzymatic systems been revealed, showing that a set of cellulolytic and non-cellulolytic glycoside hydrolases (GHs) are central players in the maintenance of the carbon cycle. Nowadays, these enzymes and the microorganisms responsible for their synthesis are constantly surveyed for their potential use in bioenergy and other industrial applications – for instance, the conversion of cellulosic biomass into biofuels. However, efficient conversion of plant biomass to fermentable sugars remains challenging (Yang *et al.*, 2009). In this context, we have evaluated the biotechnological potential of *Dyella jiangningensis* FCAV SCS01 (Xanthomonadales: Rhodanobacteraceae) through genome characterization. This bacterium was recovered from a lignocellulose-degrading microbial consortium metagenome isolated from a sugarcane crop soil, which was under mechanical harvesting and covered by decomposing straw, in an ethanol fuel plant in the state of São Paulo, Brazil (21º 19S 48º 09W – 534.1m).

*Dyella* is a gram-negative, rod-shaped bacterium that produces yellow colonies, originally found in soil, and closely related to *Frateuria*, *Rhodanobacter* and *Fulvimonas* genera from the Xanthomonadaceae family (Xie and Yokota, 2005; Zhao *et al.*, 2013). The *D. jiangningensis* species was originally isolated from the surface of weathered potassic trachyte in China, exhibiting 97.9% 16S rRNA gene sequence similarity to *Dyella japonica* (Zhao *et al.*, 2013). The different species of this genus exhibit a great diversity of biotechnologically-relevant features, such as mineral weathering, quorum-quenching activity, N-acylhomoserine lactone-degradation, thiosulfate oxidation, beta-glucosidase activity and others, mostly related to plant interaction, bio-degradation and recycling processes (An *et al.*, 2005; Chen and Chan, 2012; Bao *et al.*, 2014; Hwangbo *et al.*, 2016). In spite of their potential, this genus is still poorly studied in the genomics era. According to the Genomes Online Database, among 13 public genome sequencing projects focused on *Dyella* strains to date, three correspond to species which had their genomes completely deciphered and 10 projects remain as permanent drafts or incomplete. It is worthy of note that most sequencing projects carried out so far belong to isolates commonly found in the Asian continent, thus making the genome sequence of *D. jiangningensis* FCAV SCS01 the first from South America, and that this genome also shows an exclusive feature among its genus, related to biomass degradation.

The consortium was cultivated in BHB medium with 0.1 g/mL cycloheximide and 0.5% sugarcane straw maintained in weekly subcultures. Samples were extracted over 20 weeks for whole metagenome sequencing. A total of 69,482,643 reads (2x100 bp) were generated by the Illumina HiSeq 2500 platform using the Nextera XT DNA Sample Prep Kit and HiSeq v4 Reagent kits (Illumina), corresponding to the entire consortium metagenome. Bases presenting Phred quality scores lower than 28, sequences shorter than 30 bp and adapter sequences were removed from the datasets with Trimmomatic 0.36 (Bolger *et al.*, 2014). Filtered reads were assembled with metaSPAdes 3.10.1 (Nurk *et al.*, 2017). The full assembled metagenome was then analyzed with Kraken 0.10.5 (Wood and Salzberg, 2014) for the taxonomic assignment of sequences and ZEUSS 1.0.0 (Alvarenga *et al.*, 2017) for the retrieval of identified genome sequences. A total of 17,201,619 paired-ends and 2,719,249 single-end reads were assembled into 7 scaffolds spanning 4,758,053 bp, with an N50 of 2,446,838 and L50 of 1, representing the *Dyella jiangningensis* FCAV SCS01 genome (Table 1). These results indicate that this *Dyella* genome amounted to at least 24% of the sequenced metagenome reads. Further 16S rDNA based analysis show that the orders Burkholderiales (72.6%), Xanthomonadales (24.1%), Rhizobiales (2%), Gemmatales (0.8%), Actinomycetales (0.2%) and non classified (0.3%) compose the consortium. In spite of higher abundance, the Burkholderiales genomes were assembled in thousands of contigs, suggesting that different species may be present and making difficult the binning and assembly of each of these individuals*.*

**Table 1:** Genomic features of *Dyella jiangningensis* FCAV SCS01.

Genome feature	Value
Closed genome size (bp)	4,758,639
Coding DNA (bp)	4,164,575
G+C percentage	65.25
Contigs	7
- Longest contig	2,446,838
- Shortest contig	146,103
- Uncalled bases (Ns)	586 (0.01%)
Contig N50 (bp)	2,446,838
Total genes	4,250
Protein coding genes	4,194
- Genes with function prediction	3,193
- Hypothetical and/or unknown function	1,001
RNA genes	56
ncRNA (detected by Infernal v1.1.2)	20
CRISPR repeats (MinCED v0.1.4)	0
Pseudogenes (detected by PGAP)	54
Mobile genetic glements	19 (395,605)
- Insertion sequences (transposases)	5 (5,712bp)
- Prophage regions	2 (63,205bp)
- Genomic islands	12 (326,688bp)
Genes with assigned subsystems	1,904
Genes assigned to COGs *	3,779
Genes with Pfam domains Δ	3,449
Gene with UniProtKB	2,393
Genes with signal peptides §	675
Genes with transmembrane helices §	1,034

* Including General function prediction only (R) and Function unknown (S).

Δ Including domain of unknown function (*DUF*).

§ Hypothetical and/or unknown function genes are included.

Further analysis assisted by the SIS software (Dias *et al.*, 2012), comparisons to genomic references from other completely sequenced *Dyella* genomes (Table S1), and alignments of concordant paired reads allowed the reconstruction of two copies of the rRNA operon and the closing of the *Dyella* genome into a single scaffold containing 586 uncalled bases, representing a circular and nearly complete genome. The G+C percentage in the FCAV SCS01 genome (65.25%) and the GC Skew plot were also similar to the patterns of the currently available reference genomes. Features of the closed genome of *D. jiangningensis* FCAV SCS01 are illustrated in Figure 1.

**Figure 1:**
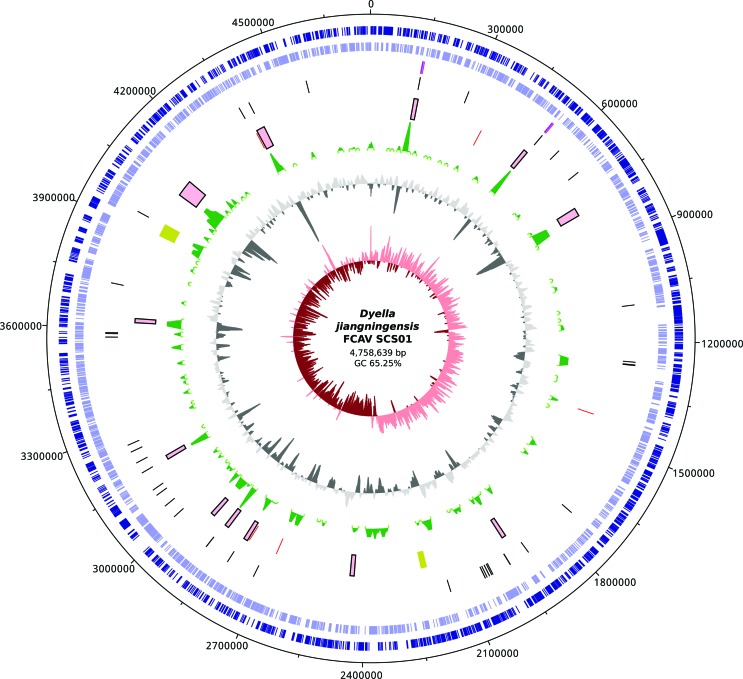
Circular genomic representation of the *Dyella jiangningensis* FCAV SCS01. The inner red circle represents GC Skew. The second level circle in gray scale represents the GC%. The third level circle in green scale represents potential anomalous regions. The purple and yellow boxes represent genomic islands and prophage-like regions, respectively. The red, black and purple bars represent insertion sequences, tRNAs and rRNA operon regions. Genes are shown in the outer blue circle, whereas genes shown on the outside of the map are transcribed clockwise, while genes on the inside are transcribed counter-clockwise.

Genome annotation performed with the RAST server (Overbeek *et al.*, 2014) and the NCBI Prokaryotic Genome Annotation Pipeline (Tatusova *et al.*, 2016) predicted a total of 4,250 genes. Among these genes, 56 encoded RNA molecules and 20 were involved in the synthesis of non-coding RNA, while 54 were pseudogenes. While hypothetical or unknown functions were assigned to 1,001 genes, 2,825 genes were identified in subsystems. The genome functional summary, characteristics and features are shown in the Table 1, and in more detail in Tables S2 and S3.

A total of 36 GHs were identified in *D. jiangningensis* FCAV SCS01 (Table 2). In addition, the FCAV SCS01 genome harbors an exclusive copy of the cellulolytic enzymes: maltodextrin glucosidase and alpha-glucosidase. These enzymes may be involved with the rapid hydrolysis of the sugarcane straw, supporting the hypothesis that this bacterium may contribute to lignocellulose decomposition.

**Table 2:** Lignocellulose decomposition-related enzymes found encoded in the *Dyella jiangningensis* FCAV SCS01 genome, and RAST based comparisons with other publicly-available *Dyella* genomes.

Enzymes	EC number	GH Family	*D. jiangningensis* FCAV SCS01	*D. jiangningensis* SBZ 3-12	*D. japonica* A8	*D. thiooxydans* ATSB10	*D. marensis* UNC178MFTsu3.1	*D. ginsengisoli* LA-4
(Hemi)cellulose			
Alpha-amylase	3.2.1.1	13, 14, 57, 119	**1**	**1**	**1**	**1**	**1**	**1**
Alpha-galactosidase	3.2.1.22	4, 27, 31, 36, 57, 97, 110	**nf**	**nf**	**nf**	**nf**	**1**	**nf**
Alpha-galactosidase percursor	3.2.1.22	4, 27, 31, 36, 57, 97, 110	**1**	**1**	**2**	**1**	**2**	**nf**
Alpha-glucosidase	3.2.1.20	4, 13, 31, 63, 97, 122	2 (*)	**1**	**nf**	**nf**	**nf**	**nf**
Alpha-glucuronidase	3.2.1.139	67	**1**	**1**	**nf**	**nf**	**nf**	**nf**
Alpha-L-fucosidase	3.2.1.51	29, 95	**3**	**3**	**4**	**nf**	**2**	**nf**
Alpha-N-acetylglucosaminidase	3.2.1.50	89	**1**	**1**	**1**	**1**	**1**	**nf**
Alpha-1,2-mannosidase	3.2.1.24	31,38,92	**7**	**8**	**4**	**3**	**4**	**3**
Alpha-xylosidase	3.2.1.177	31	**nf**	**nf**	**nf**	**1**	**nf**	**nf**
Beta-galactosidase	3.2.1.23	1, 2, 3, 35, 42, 50	**3**	**3**	**2**	**2**	**3**	**nf**
Beta-mannosidase	3.2.1.25	1, 2, 5	**2**	**2**	**2**	**1**	**1**	**1**
Beta-xylosidase	3.2.1.37	3,30,39,43,52,54	**2**	**2**	**nf**	**1**	**2**	**nf**
Endoglucanase	3.2.1.4	5, 6, 7, 9 ,12, 44, 45, 51, 74,124	**nf**	**nf**	**1**	**nf**	**nf**	**nf**
Glucoamylase	3.2.1.3	15, 97	**1**	**1**	**1**	**1**	**1**	**1**
Trehalase	3.2.1.28	13,15,37,65	**1**	**1**	**1**	**2**	**1**	**2**
Malto-oligosyltrehalose trehalohydrolase	3.2.1.141	13	**1**	**1**	**nf**	**nf**	**nf**	**nf**
Maltodextrin glucosidase	3.2.1.20	4, 13	2 (*)	**1**	**nf**	**nf**	**nf**	**nf**
alpha-N-acetylgalactosaminidase	3.2.1.49	36	**1**	**1**	**1**	**nf**	**1**	**nf**
Xylanase	3.2.1.8	5, 8, 10, 11, 43	**1**	**1**	**nf**	**2**	**1**	**nf**
Endo-1,4-beta-xylanase A precursor	3.2.1.8	5, 8, 10, 11, 43	**nf**	**nf**	**nf**	**nf**	**1**	**nf**
Chitin								
Beta-hexosaminidase	3.2.1.52	3,5,18,20,84,116	**4**	**3**	**4**	**1**	**3**	**1**
Chitinase	3.2.1.14	18, 19	**2**	**2**	**2**	**1**	**3**	**1**
**TOTAL GHs**			**36**	**34**	**26**	**19**	**28**	10

* *D. jiangningensis* FCAV SCS01 carry an exclusive copy.

“nf” means not found

Few mobile genetic elements (MGE) clusters were identified using the current protocol by (Alvarenga *et al.*, 2018). The MGEs are spread over the entire genome, and are related to five insertion sequence regions, mostly belonging to the IS2 ssgr IS51 family, two incomplete and degenerated prophage regions and 12 potential genomic islands (GIs). In general, the GIs encode genes related to the general metabolism; nonetheless, antibiotic and multidrug resistance, type IV secretion systems and others not directly related to biomass degradation were identified (for a full list please see Table S4). antiSMASH 4.0.0rc1 (Blin *et al.*, 2017) was used for predicting gene clusters potentially involved in the biosynthesis of secondary metabolites, and uncovered clusters coding for novel aryl-polyene, bacteriocin, and terpene molecules, in addition to the products of a hybrid non-ribosomal peptide synthetase and polyketide synthase pathway. However, the predicted gene clusters presented low or no similarity to biosynthetic clusters described for known molecules, suggesting that this strain might also be a source of novel chemicals. It is worthy to mention that none of these clusters were included in the MGEs clusters.

Comparative analyses among closely related and different *Dyella* species indicated that the FCAV SCS01 protein coding regions show 90.77%, 78.37%, 70.35%, 65.60%, 65.35% of identity to *D. jiangningensis* SBZ 3-12, *D. japonica* A8, *D. marensis* UNC178MFTsu3.1, *D. thiooxydans* ATSB10 and *D. ginsengisoli* LA-4, respectively. Moreover, the 16S rRNA gene in this strain is 99% identical to that of *D. jiangningensis* SBZ 3-12, thus corroborating the taxonomic identification. In addition, these species share 2,147 orthologous clusters (Figure 2A). However, despite the high number of orthologous clusters, genome collinearity is not conserved among the completely sequenced species, whereas several inversions and translocations were observed (Figure 2B).

**Figure 2:**
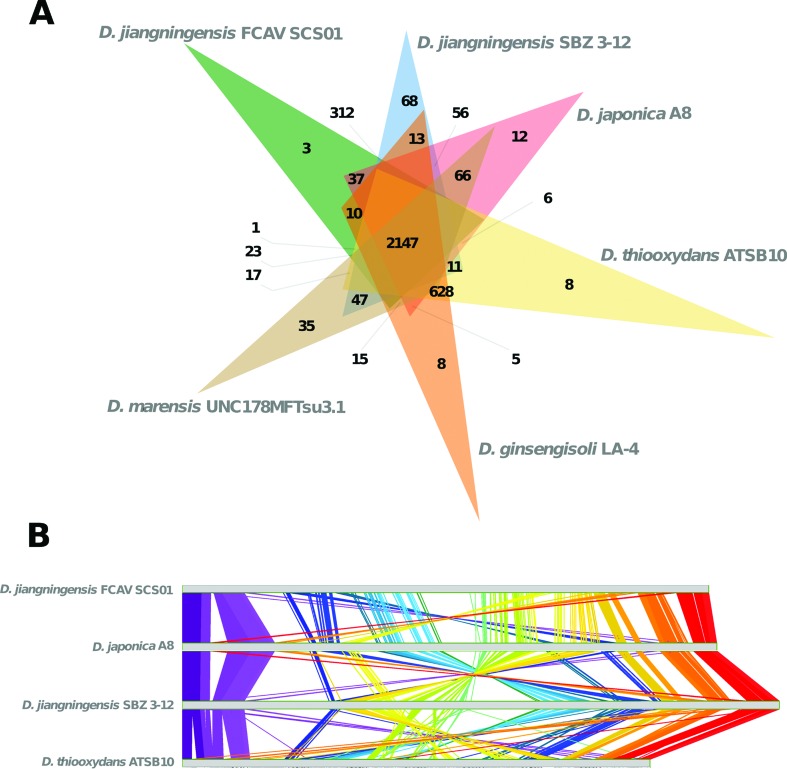
Comparative genomics of *Dyella jiangningensis* FCAV SCS01 against other publicly available *Dyella* genomes. (A) Venn diagram showing shared orthologous clusters generated by OrthoVenn webservice (Wang *et al.*, 2015). The genus forms 5,029 clusters, 4,895 orthologous clusters (containing at least two species) and 1,951 single-copy gene clusters; (B) Syntenic regions between *Dyella* genomes generated by M-GCAT (Treangen and Messeguer 2006). For the alignment only complete genomes were considered (please refer to Table S1). The *dnaA* gene was used as starting point for the alignment.

Despite finding only three orthologous clusters related to unknown proteins exclusive to FCAV SCS01, this genome also harbors 372 unique genes. Most of these genes correspond to hypothetical proteins (265). However, remarkable features, such as chemotaxis-related proteins, chitosanase, cytochrome c oxidase polypeptide I, II, III, and IV, and an almost complete copy of a conjugative transfer operon (*traB*,*C*,*D*,*E*,*F*,*G*,*I*,*J*,*L*) were identified. Potential enzymes related to biomass degradation, like hydrolase, glycosyltransferase, and maltose transporter and maltose-specific TonB-dependent receptor, were also identified (a full list of the unique features is shown in Table S5).

Overall, the *Dyella jiangningensis* FCAV SCS01 genome presents several relevant features, including enzymes which can be exploited for bioenergy production, therefore supporting its use in a number of biotechnological applications. Since not all biomass degradation related enzymes were found in the *Dyella* genome, it is plausible that the microorganisms present in the consortium might act synergistically for the biomass degradation. Therefore, this hypothesis, and the possible metabolic potential and ecological interactions between these microorganisms in the consortium will be evaluated in a future work. The annotated sequences of *Dyella jiangningensis* FCAV SCS01 have been deposited in the DDBJ/EMBL/GenBank database under the accession number NFZS00000000. The version described in this paper is the first version.

## Supplementary material

The following online material is available for this article:

Table S1 - Publicly-available *Dyella* genomes used in this study for comparative genomics.

Table S2 - Subsystems found in *Dyella jiangningensis* FCAV SCS01 and other publicly available *Dyella* genomes.

Table S3 - Clusters of Orthologous Groups (COGs) found in *Dyella jiangningensis* FCAV SCS01 and other publicly available *Dyella* genomes.

Table S4 - Gene content of each MGE identified in *Dyella jiangningensis* FCAV SCS01 complete genome.

Table S5 - The 371 exclusive genes found in *Dyella jiangningensis* FCAV SCS01.
