# Supplementary material for: The genome sequence of Dyella jiangningensis FCAV SCS01 from a lignocellulose-decomposing microbial consortium metagenome reveals potential for biotechnological applications
Source: Genet Mol Biol. 2018 May 14;41(2):507–13. doi: 10.1590/1678-4685-GMB-2017-0155 (PMC6082245; doi:10.1590/1678-4685-GMB-2017-0155)
Supplement: Supplementary file 1 [file 1415-4757-GMB-10159016784685GMB20170155-s001.pdf]

Supplementary Material to "The genome sequence of *Dyella jiangningensis* FCAV SCS01 from a lignocellulose-decomposing microbial consortium metagenome reveals potential for biotechnological applications"

Table S1 - Publicly-available *Dyella* genomes used in this study for comparative genomics.

Genome	Size (bp)	CDS	Clusters	Singletons	Isolation source
D. jiangningensis **FCAV SCS01**	4,758,639	4,194	2,789	371	Sugarcane decomposing straw; Brazil: Sao Paulo
D. jiangningensis **SBZ 3-12**	5,396,991	4,827	3975	474	Surfaces of weathered potassic trachyte; China: Nanjing
D. japonica **A8**	4,831,185	4,133	3437	639	Soil; Malaysia: Rimba Ilmu
D. thiooxydans **ATSB10**	4,227,172	3,777	3414	320	Rhizosphere soil of sunflower (*Helianthus annuus* L.); South Korea: Cheongwon
D. marensis **UNC178MFTsu3.1** (draft)	5,005,584	4,367	3192	1,070	Plant associated metagenome, location not specified
D. ginsengisoli **LA-4** (draft)	4,546,859	4,054	3420	598	Activated sludge; China: Dalin
