# Supplementary material for: The genome sequence of Dyella jiangningensis FCAV SCS01 from a lignocellulose-decomposing microbial consortium metagenome reveals potential for biotechnological applications
Source: Genet Mol Biol. 2018 May 14;41(2):507–13. doi: 10.1590/1678-4685-GMB-2017-0155 (PMC6082245; doi:10.1590/1678-4685-GMB-2017-0155)
Supplement: Supplementary file 2 [file 1415-4757-GMB-10159016784685GMB20170155-s002.pdf]

Supplementary Material to "The genome sequence of *Dyella jiangningensis* FCAV SCS01 from a lignocellulose-decomposing microbial consortium metagenome reveals potential for biotechnological applications"

Table S2 - Subsystems found in Dyella jiangningensis FCAV SCS01 and other publicly available Dyella genomes.

Description	FCAV SCS01	LA-4	A8	SBZ 3-12	UNC178MFTsu3	ATSB10
Cofactors, Vitamins, Prosthetic Groups, Pigments	258	228	257	286	249	234
Cell Wall and Capsule	127	110	114	143	156	111
Virulence, Disease and Defense	126	108	130	152	118	90
Potassium metabolism	16	20	16	17	17	20
Miscellaneous	54	27	54	57	52	42
Phages, Prophages, Transposable elements, Plasmids	1	0	2	5	29	1
Membrane Transport	148	127	166	172	143	125
Iron acquisition and metabolism	17	14	18	20	54	15
RNA Metabolism	162	157	161	176	164	162
Nucleosides and Nucleotides	66	82	77	95	82	82
Protein Metabolism	278	273	272	292	269	276
Cell Division and Cell Cycle	32	36	32	34	32	32
Motility and Chemotaxis	102	126	99	103	93	103
Regulation and Cell signaling	48	48	53	56	66	41
Secondary Metabolism	5	5	5	5	9	5
DNA Metabolism	103	106	87	118	104	101
Fatty Acids, Lipids, and Isoprenoids	141	160	155	154	130	164
Nitrogen Metabolism	25	22	21	52	21	22
Dormancy and Sporulation	1	1	1	1	1	2
Respiration	143	122	138	145	115	127
Stress Response	132	160	120	171	122	156
Metabolism of Aromatic Compounds	24	31	25	33	29	27
Amino Acids and Derivatives	371	335	362	432	385	335
Sulfur Metabolism	32	24	35	37	33	26
Phosphorus Metabolism	50	45	55	51	45	42
Carbohydrates	363	266	301	404	325	286
Not in subsystems	2288	2258	2273	2687	2473	1988
