# Supplementary material for: The genome sequence of Dyella jiangningensis FCAV SCS01 from a lignocellulose-decomposing microbial consortium metagenome reveals potential for biotechnological applications
Source: Genet Mol Biol. 2018 May 14;41(2):507–13. doi: 10.1590/1678-4685-GMB-2017-0155 (PMC6082245; doi:10.1590/1678-4685-GMB-2017-0155)
Supplement: Supplementary file 3 [file 1415-4757-GMB-10159016784685GMB20170155-s003.pdf]

Supplementary Material to "The genome sequence of *Dyella jiangningensis* FCAV SCS01 from a lignocellulose-decomposing microbial consortium metagenome reveals potential for biotechnological applications"

Table S3 - Clusters of Orthologous Groups **(**COGs) found in *Dyella jiangningensis* FCAV SCS01 and other publicly available *Dyella* genomes. The values are followed by their percentages in parentheses.

Code	Description	FCAV SCS01	LA-4	A8	SBZ 3-12	UNC178MFT su3	ATSB10
A	**RNA processing and modification**	**1** (0.02)	**1** (0.02)	**1** (0.02)	**1** (0.02)	**1** (0.02)	**1** (0.03)
B	**Chromatin structure and dynamics**	**1** (0.02)	**1** (0.02)	**1** (0.02)	**2** (0.04)	**2** (0.04)	**1** (0.03)
C	**Energy production and conversion**	**205** (4.88)	**194** (4.79)	**215** (5.20)	**235** (4.87)	**201** (4.62)	**195** (5.16)
D	**Cell cycle control, cell division, chromosome partitioning**	**34** (0.81)	**36** (0.89)	**36** (0.87)	**38** (0.79)	**32** (0.73)	**31** (0.82)
E	**Amino acid transport and metabolism**	**258** (6.15)	**241** (5.94)	**250** (6.05)	**295** (6.11)	**257** (5.89)	**239** (6.33)
F	**Nucleotide transport and metabolism**	**61** (1.45)	**65** (1.60)	**62** (1.50)	**72** (1.49)	**64** (1.47)	**61** (1.62)
G	**Carbohydrate transport and metabolism**	**217** (5.17)	**155** (3.82)	**183** (4.43)	**236** (4.89)	**220** (5.04)	**175** (4.63)
H	**Coenzyme transport and metabolism**	**146** (3.48)	**122** (3.01)	**156** (3.77)	**166** (3.44)	**148** (3.39)	**121** (3.20)
I	**Lipid transport and metabolism**	**132** (3.15)	**144** (3.55)	**140** (3.39)	**156** (3.23)	**124** (2.84)	**144** (3.81)
J	**Translation, ribosomal structure and biogenesis**	**181** (4.32)	**181** (4.46)	**176** (4.26)	**189** (3.92)	**185** (4.24)	**179** (4.74)
K	**Transcription**	**258** (6.15)	**230** (5.67)	**262** (6.34)	**291** (6.03)	**304** (6.96)	**227** (6.01)
L	**Replication, recombination and repair**	**133** (3.17)	**164** (4.05)	**125** (3.02)	**157** (3.25)	**130** (2.98)	**141** (3.73)
M	**Cell wall/membrane/envelope biogenesis**	**259** (6.18)	**238** (5.87)	**262** (6.34)	**308** (6.38)	**265** (6.07)	**247** (6.54)
N	**Cell motility**	**125**	**138**	**127**	**138**	**133**	**110**

Code	Description	FCAV SCS01	LA-4	A8	SBZ 3-12	UNC178MFT su3	ATSB10
		(2.98)	(3.40)	(3.07)	(2.86)	(3.05)	(2.91)
O	**Posttranslational modification, protein turnover, chaperones**	**170** (4.05)	**168** (4.14)	**176** (4.26)	**192** (3.98)	**166** (3.80)	**162** (4.29)
P	**Inorganic ion transport and metabolism**	**191** (4.55)	**150** (3.70)	**171** (4.14)	**205** (4.25)	**191** (4.37)	**147** (3.89)
Q	**Secondary metabolites biosynthesis, transport and catabolism**	**103** (2.46)	**95** (2.34)	**86** (2.08)	**124** (2.57)	**87** (1.99)	**87** (2.30)
R	**General function prediction only**	**454** (10.82)	**406** (10.01)	**424** (10.26)	**515** (10.67)	**438** (10.03)	**391** (10.36)
S	**Function unknown**	**377** (8.99)	**327** (8.07)	**380** (9.19)	**407** (8.43)	**400** (9.16)	**327** (8.66)
T	**Signal transduction mechanisms**	**224** (5.34)	**243** (5.99)	**223** (5.40)	**253** (5.24)	**219** (5.01)	**237** (6.27)
U	**Intracellular trafficking, secretion, and vesicular transport**	**165** (3.93)	**138** (3.40)	**157** (3.80)	**177** (3.67)	**165** (3.78)	**109** (2.89)
V	**Defense mechanisms**	**79** (1.88)	**95** (2.34)	**88** (2.13)	**99** (2.05)	**89** (2.04)	**88** (2.33)
W	**Extracellular structures**	**4** (0.09)	**0** (0.00)	**7** (0.17)	**2** (0.04)	**5** (0.11)	**0** (0.00)
Z	**Cytoskeleton**	**1** (0.02)	**2** (0.05)	**1** (0.02)	**1** (0.02)	**1** (0.02)	**1** (0.03)
-	**Not in COGs**	**415** (9.89)	**520** (12.83)	**424** (10.26)	**568** (11.77)	**540** (12.37)	**356** (9.43)
