# Supplementary material for: The genome sequence of Dyella jiangningensis FCAV SCS01 from a lignocellulose-decomposing microbial consortium metagenome reveals potential for biotechnological applications
Source: Genet Mol Biol. 2018 May 14;41(2):507–13. doi: 10.1590/1678-4685-GMB-2017-0155 (PMC6082245; doi:10.1590/1678-4685-GMB-2017-0155)
Supplement: Supplementary file 4 [file 1415-4757-GMB-10159016784685GMB20170155-s004.pdf]

Supplementary Material to "The genome sequence of *Dyella jiangningensis* FCAV SCS01 from a lignocellulose-decomposing microbial consortium metagenome reveals potential for biotechnological applications"

Table S4 - Gene content of each MGE identified in *Dyella jiangningensis* FCAV SCS01 complete genome.

Type	Coordinates	Feature
Prophage 1 (2197172..2217675)	2197172..2197636	Transcriptional regulator, AsnC family
	2197882..2198856	Sigma-54-dependent transcriptional activator
	c(2198902..2199114)	hypothetical protein
	2199118..2199333	hypothetical protein
	2199367..2200962	Phage tail sheath protein FI
	2200973..2201500	hypothetical protein
	2201504..2202250	hypothetical protein
	2202742..2202960	hypothetical protein
	2202957..2204201	Glutamine synthetase adenylyltransferase
	2204198..2206384	ATPase, AAA family
	c(2206412..2207074)	hypothetical protein
	2207030..2208151	hypothetical protein
	2208181..2210298	hypothetical protein
	2210534..2211271	hypothetical protein
	2211268..2211924	hypothetical protein
	2211924..2212244	hypothetical protein
	2212500..2213663	hypothetical protein
	2213660..2214166	VgrG protein
	c(2214195..2214497)	hypothetical protein

Type	Coordinates	Feature
	2214509..2214880	hypothetical protein
	2214877..2217675	hypothetical protein
	2217672..2220788	hypothetical protein
Prophage 2 (3910255..3952957)	3910255..3911214	Bores hole in peptidoglycan layer allowing type IV secretion complex assembly to occur (VirB1)
	3911421..3911840	Major pilus subunit of type IV secretion complex, VirB2
	3911932..3912141	Inner membrane protein forms channel for type IV secretion of T-DNA complex, VirB3
	3912247..3914712	ATPase provides energy for both assembly of type IV secretion complex and secretion of T-DNA complex (VirB4)
	3914745..3915527	hypothetical protein
	3915611..3916717	Inner membrane protein of type IV secretion of T-DNA complex, VirB6
	3917866..3918093	hypothetical protein
	3918904..3919296	hypothetical protein
	3919346..3920566	Phage endolysin
	3920709..3921245	hypothetical protein
	3921415..3921939	hypothetical protein
	3921936..3923612	hypothetical protein
	3923705..3925384	Type IV secretion system protein VirD4
	3925512..3926990	Putative membrane protein
	3927049..3928419	Glycolate dehydrogenase (EC 1.1.99.14), subunit GlcD
	c(3928540..3929178)	hypothetical protein
	c(3929193..3929519)	hypothetical protein
	c(3929558..3930922)	Tryptophanyl-tRNA synthetase (EC 6.1.1.2)
	c(3930957..3931871)	Arginase (EC 3.5.3.1)
	c(3931881..3932237)	Rhodanese domain protein
	c(3932486..3933199)	Methionyl-tRNA formyltransferase (EC 2.1.2.9)
	c(3933246..3934238)	hypothetical protein

Type	Coordinates	Feature
	c(3935253..3935459)	hypothetical protein
	c(3935869..3936051)	hypothetical protein
	c(3936367..3936570)	hypothetical protein
	c(3939066..3939959)	DNA primase (EC 2.7.7.-), phage-associated
	c(3939956..3940204)	hypothetical protein
	c(3940201..3940458)	hypothetical protein
	c(3941322..3942002)	Phage tail assembly protein I
	c(3941999..3942208)	hypothetical protein
	c(3942205..3942408)	hypothetical protein
	3944289..3944444	hypothetical protein
	c(3944525..3944890)	hypothetical protein
	c(3945421..3946680)	Integrase
	c(3946829..3946904)	tRNA-Thr-TGT
	c(3946940..3947662)	hypothetical protein
	c(3947699..3948457)	Pantothenate kinase type III, CoaX-like (EC 2.7.1.33)
	c(3948454..3949404)	Biotin operon repressor / Biotin-protein ligase (EC 6.3.4.15)
	3949768..3949989	hypothetical protein
	3950080..3950769	Inner membrane protein
	c(3950881..3952203)	Sensor protein PhoQ (EC 2.7.13.3)
	c(3952265..3952957)	Two-component system regulatory protein
Genome island 1 (133000..157099)	133018..133827	Exodeoxyribonuclease III (EC 3.1.11.2)
	133817..135142	AmpG permease
	135229..135528	hypothetical protein
	c(135525..136643)	Anhydro-N-acetylmuramic acid kinase (EC 2.7.1.-)
	c(136650..137933)	Peptidase, M23/M37 family

Type	Coordinates	Feature
	137946..138062	hypothetical protein
	138273..139484	Tyrosyl-tRNA synthetase (EC 6.1.1.1)
	139940..141473	16S rRNA
	141621..141697	tRNA-Ile-GAT
	141762..141837	tRNA-Ala-TGC
	142039..144908	23S rRNA
	145083..145201	5S rRNA
	145656..146339	FIG056164: rhomboid family serine protease
	146349..146672	hypothetical protein
	c(146678..147802)	hypothetical protein
	c(147889..148611)	GMP synthase [glutamine-hydrolyzing] (EC 6.3.5.2)
	c(148651..150462)	Type II secretory pathway, ATPase PulE/Tfp pilus assembly pathway, ATPase PilB
	150557..151468	Glycyl-tRNA synthetase alpha chain (EC 6.1.1.14)
	151465..153612	Glycyl-tRNA synthetase beta chain (EC 6.1.1.14)
	c(153672..154238)	hypothetical protein
	154590..157481	TonB-dependent receptor
Genome island 2 (522500..546599)	522715..522837	hypothetical protein
	c(522846..523028)	hypothetical protein
	523023..524258	Acetylornithine aminotransferase (EC 2.6.1.11)
	c(524452..525327)	Potassium voltage-gated channel subfamily KQT; possible potassium channel, VIC family
	c(525403..526008)	hypothetical protein
	c(526013..527299)	Glutamate-1-semialdehyde aminotransferase (EC 5.4.3.8)
	c(527308..527931)	Thiamin-phosphate pyrophosphorylase (EC 2.5.1.3)
	528069..528224	Rubredoxin
	528344..528553	hypothetical protein

Type	Coordinates	Feature
	529287..530820	16S rRNA
	530968..531044	tRNA-Ile-GAT
	531109..531184	tRNA-Ala-TGC
	531386..534257	23S rRNA
	534432..534550	5S rRNA
	535360..535920	putative membrane protein
	536004..537503	Glycerol kinase (EC 2.7.1.30)
	c(537556..538491)	PfkB
	538754..539800	Rod shape-determining protein MreB
	539827..540813	Rod shape-determining protein MreC
	540810..541298	Rod shape-determining protein MreD
	541303..543306	Penicillin-binding protein 2 (PBP-2)
	543303..544421	Rod shape-determining protein RodA
	544574..545587	Membrane-bound lytic murein transglycosylase B precursor (EC 3.2.1.-)
	545584..546516	Rare lipoprotein A precursor
	546576..547805	D-alanyl-D-alanine carboxypeptidase (EC 3.4.16.4)
Genome island 3 (768000..805599)	767435..768805	Cystathionine beta-synthase (EC 4.2.1.22)
	c(768785..768970)	hypothetical protein
	768941..770770	Glucosamine--fructose-6-phosphate aminotransferase [isomerizing] (EC 2.6.1.16)
	c(770832..772244)	Mannose-1-phosphate guanylyltransferase (GDP) (EC 2.7.7.22)
	c(772248..773183)	dTDP-4-dehydrorhamnose reductase (EC 1.1.1.133)
	c(773180..773737)	dTDP-4-dehydrorhamnose 3,5-epimerase (EC 5.1.3.13)
	c(773734..774621)	Glucose-1-phosphate thymidylyltransferase (EC 2.7.7.24)
	c(774618..775586)	dTDP-glucose 4,6-dehydratase (EC 4.2.1.46)
	775887..777053	Cystathionine gamma-lyase (EC 4.4.1.1)

Type	Coordinates	Feature
	c(777318..777482)	hypothetical protein
	777806..779290	Teichoic acid export ATP-binding protein TagH (EC 3.6.3.40)
	779505..782051	Glycosyltransferase
	782846..784396	hypothetical protein
	c(784505..785038)	methyltransferase
	785778..787013	Lipopolysaccharide modification acyltransferase
	c(787035..787421)	UDP-glucose 4-epimerase (EC 5.1.3.2)
	c(789243..791696)	Glycosyltransferase
	c(791972..792274)	Mobile element protein
	c(792763..794865)	hypothetical protein
	c(794927..796216)	UDP-glucose dehydrogenase (EC 1.1.1.22)
	c(796438..797049)	hypothetical protein
	c(797031..797315)	hypothetical protein
	c(797413..798369)	dolichol-phosphate mannosyltransferase
	c(798376..799326)	dTDP-glucose 4,6-dehydratase (EC 4.2.1.46)
	c(799675..801030)	Phosphomannomutase (EC 5.4.2.8)
	c(801037..801978)	Electron transfer flavoprotein, alpha subunit
	c(802000..802746)	Electron transfer flavoprotein, beta subunit
	c(802856..804979)	Glycosyltransferase
	c(804948..806549)	Electron transfer flavoprotein-ubiquinone oxidoreductase (EC 1.5.5.1)
Genome island 4 (1930000..1947599)	c(1928609..1930498)	Topoisomerase IV subunit B (EC 5.99.1.-)
	c(1930680..1932482)	Xaa-Pro aminopeptidase (EC 3.4.11.9)
	1932489..1932704	hypothetical protein
	1932701..1933675	Cytochrome O ubiquinol oxidase subunit II (EC 1.10.3.-)

Type	Coordinates	Feature
	j(1933679..1934557,1934559..1935704)	Cytochrome O ubiquinol oxidase subunit I (EC 1.10.3.-)
	1935708..1936343	Cytochrome O ubiquinol oxidase subunit III (EC 1.10.3.-)
	1936343..1936720	Cytochrome O ubiquinol oxidase subunit IV (EC 1.10.3.-)
	1937053..1938009	Glycosyl transferase, group 2 family protein
	1938009..1939154	D amino acid oxidase (DAO) family (EC 1.4.3.3)
	1939151..1939945	hypothetical protein
	1940772..1941128	Quaternary ammonium compound-resistance protein SugE
	1941140..1941946	glycosyltransferase(EC:2.4.-)
	1941993..1943540	Conjugative transfer protein TrbL
	1943761..1945431	CTP synthase (EC 6.3.4.2)
	1945428..1946261	2-Keto-3-deoxy-D-manno-octulosonate-8-phosphate synthase (EC 2.5.1.55)
	j(1946355..1947515,1947519..1947524,1947524..1947670)	Enolase (EC 4.2.1.11)
Genome island 5 (2438000..2460099)	2437265..2438410	L-lactate dehydrogenase (EC 1.1.2.3)
	c(2439236..2439586)	L-fucose mutarotase, type 2
	c(2439583..2440884)	Fucose permease
	2441113..2441844	Transcriptional regulator, IclR family
	c(2442008..2442298)	hypothetical protein
	c(2443129..2444115)	hypothetical protein
	c(2444366..2444644)	hypothetical protein
	c(2444789..2445283)	VgrG protein
	c2446911..2447231	D-alanyl-D-alanine carboxypeptidase (EC 3.4.16.4)
	2447607..2447918	sensory box histidine kinase/response regulator
	c(2448011..2450038)	hypothetical protein
	c(2450061..2451554)	Circadian clock protein KaiC

Type	Coordinates	Feature
	2452680..2455697	TonB-dependent receptor
	2456199..2457104	6-phosphogluconate dehydrogenase, decarboxylating (EC 1.1.1.44)
	c(2457123..2459255)	Transketolase (EC 2.2.1.1)
	c(2459256..2460347)	Transaldolase (EC 2.2.1.2)
Genome island 6 (2799000..2821599)	c(2799009..2801243)	Type II secretory pathway, ATPase PulE/Tfp pilus assembly pathway, ATPase PilB
	c(2801260..2802372)	UDP-N-acetylglucosamine 2-epimerase (EC 5.1.3.14)
	c(2802818..2802970)	hypothetical protein
	2803030..2804580	hypothetical protein
	2804826..2805206	
	c(2805249..2806151)	putative membrane protein
	2807034..2807231	hypothetical protein
	2807370..2807681	hypothetical protein
	2807822..2808187	hypothetical protein
	2808422..2808547	hypothetical protein
	2809283..2810599	Flagellar hook-length control protein FliK
	c(2810697..2811694)	Mobile element protein
	c(2811658..2811771)	Mobile element protein
	c(2811972..2812056)	tRNA-Leu-GAG
	c(2812068..2812514)	Preprotein translocase subunit SecG (TC 3.A.5.1.1)
	c(2812539..2813288)	Triosephosphate isomerase (EC 5.3.1.1)
	2813411..2814223	oxidoreductase, short-chain dehydrogenase/reductase family
	c(2814220..2814672)	hypothetical protein
	c(2815551..2816486)	Oxidoreductase
	c(2816690..2818042)	Phosphoglucosamine mutase (EC 5.4.2.10)
	c(2818101..2819009)	Dihydropteroate synthase (EC 2.5.1.15)

Type	Coordinates	Feature
	c(2819034..2819594)	3'-to-5' oligoribonuclease (orn)
	2819687..2821135	Exodeoxyribonuclease I (EC 3.1.11.1)
	2821145..2821831	hypothetical protein
Genome island 7 (2872000..2896099)	c(2871604..2872617)	Ribosomal large subunit pseudouridine synthase C (EC 4.2.1.70)
	2872969..2876046	Ribonuclease E (EC 3.1.26.12)
	c(2876120..2876773)	DNA-binding response regulator, LuxR family
	c(2876924..2877166)	hypothetical protein
	c(2877573..2878868)	virulence-associated protein E
	c(2880654..2880866)	hypothetical protein
	c(2882483..2882665)	hypothetical protein
	c(2884842..2886068)	Integrase
	c(2886252..2886338)	tRNA-Leu-TAA
	c(2886438..2886511)	tRNA-Cys-GCA
	c(2886596..2886671)	tRNA-Gly-GCC
	2886943..2887644	DUF209:Cupin domain
	2887724..2888671	Beta-lactamase related protein
	c(2888684..2889295)	Transcriptional regulator, TetR family
	2889365..2890639	putative membrane transport protein
	c(2890640..2890990)	Protein secretion chaperonin CsaA
	c(2890987..2892285)	Oxidoreductase
	c(2892333..2894726)	Multimodular transpeptidase-transglycosylase (EC 2.4.1.129) (EC 3.4.-.-)
	c(2894776..2900805)	Large extracellular alpha-helical protein
Genome island 8 (2930000..2952599)	2929968..2931563	Sensor histidine kinase
	c(2931957..2932370)	Ferric uptake regulation protein FUR
	2932448..2932897	Outer membrane lipoprotein SmpA, a component of the essential YaeT outer-membrane protein assembly complex

Type	Coordinates	Feature
	c(2932913..2933194)	UPF0125 protein yfjF
	c(2933187..2933618)	Putative oligoketide cyclase/lipid transport protein, similarity with yeast ubiquinone-binding protein YOL008W
	2933702..2934193	tmRNA-binding protein SmpB
	c(2934185..2934697)	Histone acetyltransferase HPA2 and related acetyltransferases
	2935288..2935428	hypothetical protein
	c(2935566..2935715)	hypothetical protein
	c(2936272..2936934)	Transcriptional regulator, ArsR family
	2937233..2937568	(AF179595) Vco33
	c(2941063..2941491)	hypothetical protein
	c(2941598..2941846)	hypothetical protein
	c(2942076..2942387)	hypothetical protein
	(2942794..2942949)	hypothetical protein
	c(2943083..2943844)	ABC-type multidrug transport system, permease component
	c(2943841..2944788)	ABC-type multidrug transport system, ATPase component
	2945119..2945394	FrmR: Negative transcriptional regulator of formaldehyde detoxification operon
	2945451..2946560	S-(hydroxymethyl)glutathione dehydrogenase (EC 1.1.1.284)
	c(2946784..2946978)	hypothetical protein
	c(2947272..2947394)	hypothetical protein
	c(2947808..2948836)	Extracellular protease
	c(2948944..2949135)	hypothetical protein
	2949176..2949667	RNA polymerase sigma-54 factor RpoN
	2949664..2950422	hypothetical protein
	c(2950551..2950886)	hypothetical protein
	c(2951062..2951448)	hypothetical protein
	2951694..2954009	TonB-dependent receptor

Type	Coordinates	Feature
Genome island 9 (3162000..3183099)	3161423..3162349	Transcriptional regulator, ArsR family / Methyltransferase fusion
	3162361..3163440	5-methyltetrahydrofolate--homocysteine methyltransferase (EC 2.1.1.13)
	3163447..3166131	5-methyltetrahydrofolate--homocysteine methyltransferase (EC 2.1.1.13)
	c(3166255..3166596)	hypothetical protein
	3166730..3166804	tRNA-Val-CAC
	3167982..3168098	hypothetical protein
	c(3168860..3174796)	Flagellar hook-length control protein FliK
	c(3174996..3175301)	hypothetical protein
	3176232..3176654	hypothetical protein
	c(3176933..3178525)	hypothetical protein
	c(3178527..3178976)	hypothetical protein
	c(3179157..3179318)	hypothetical protein
	3179307..3181760	ferric enterobactin receptor
	c(3181785..3181940)	hypothetical protein
	3181929..3185360	Exodeoxyribonuclease V gamma chain (EC 3.1.11.5)
Genome island 10 (3610000..3631099)	c(3609968..3611005)	Membrane-bound lytic murein transglycosylase D precursor (EC 3.2.1.-)
	3611656..3612999	tRNA-i(6)A37 methylthiotransferase
	3613207..3614193	Phosphate starvation-inducible protein PhoH, predicted ATPase
	3614190..3614657	Metal-dependent hydrolase YbeY, involved in rRNA and/or ribosome maturation and assembly
	3614985..3615767	Magnesium and cobalt efflux protein CorC
	3615794..3617047	hypothetical protein
	3617142..3618110	Magnesium and cobalt transport protein CorA
	3618778..3620472	TPR domain protein
	c(3620539..3622608)	alanyl dipeptidyl peptidase
	3623108..3623566	Type IV pilin PilA

Type	Coordinates	Feature
	3623732..3625801	TPR-repeat-containing protein
	3625798..3627042	polysaccharide biosynthesis protein
	c(3630164..3631789)	Asparagine synthetase [glutamine-hydrolyzing] (EC 6.3.5.4)
Genome island 11 (3907000..3931599) (overlaps with prophage 2)	3906223..3907158	Inner membrane protein forms channel for type IV secretion of T-DNA complex, VirB8
	3907158..3907925	Outer membrane and periplasm component of type IV secretion of T-DNA complex, has secretin-like domain, VirB9
	3907922..3909184	Inner membrane protein forms channel for type IV secretion of T-DNA complex (VirB10)
	3909203..3910243	ATPase provides energy for both assembly of type IV secretion complex and secretion of T-DNA complex (VirB11)
	3910255..3911214	Bores hole in peptidoglycan layer allowing type IV secretion complex assembly to occur (VirB1)
	3911421..3911840	Major pilus subunit of type IV secretion complex, VirB2
	3911932..3912141	Inner membrane protein forms channel for type IV secretion of T-DNA complex, VirB3
	3912247..3914712	ATPase provides energy for both assembly of type IV secretion complex and secretion of T-DNA complex (VirB4)
	3914745..3915527	hypothetical protein
	3915611..3916717	Inner membrane protein of type IV secretion of T-DNA complex, VirB6
	3917866..3918093	hypothetical protein
	3918904..3919296	hypothetical protein
	3919346..3920566	Phage endolysin
	3920709..3921245	hypothetical protein
	3921415..3921939	hypothetical protein
	3921936..3923612	hypothetical protein
	3923705..3925384	Type IV secretion system protein VirD4
	3925512..3926990	Putative membrane protein
	3927049..3928419	Glycolate dehydrogenase (EC 1.1.99.14), subunit GlcD
	c(3928540..3929178)	hypothetical protein
	c(3929193..3929519)	hypothetical protein
	c(3929558..3930922)	Tryptophanyl-tRNA synthetase (EC 6.1.1.2)

Type	Coordinates	Feature
	c(3930957..3931871)	Arginase (EC 3.5.3.1)
Genome island 12 (4041500..4103099)	4041407..4041895	hypothetical protein
	4041971..4042582	Integral membrane protein
	c(4042635..4043327)	hypothetical protein
	c(4043769..4044611)	hypothetical protein
	c(4045110..4045481)	hypothetical protein
	c(4045641..4046588)	hypothetical protein
	4046782..4047780	putative transmembrane protein
	4048022..4048504	hypothetical protein
	4050258..4051595	hypothetical protein
	c(4052878..4054620)	N-acyl-D-amino-acid deacylase precursor (EC 3.5.1.81)
	c(4054813..4056405)	hypothetical protein
	c(4056589..4057083)	hypothetical protein
	c(4057296..4059275)	Adenylate cyclase (EC 4.6.1.1)
	4059279..4059446	hypothetical protein
	4059599..4060573	Outer membrane protein
	4061026..4065894	autotransporter
	c(4065918..4066160)	hypothetical protein
	c(4066343..4066516)	hypothetical protein
	4069113..4070183	D-alanyl-D-alanine carboxypeptidase (EC 3.4.16.4)
	c(4070180..4070302)	hypothetical protein
	4070728..4070886	hypothetical protein
	c(4071548..4071895)	hypothetical protein
	c(4073729..4073899)	hypothetical protein
	4074598..4074831	hypothetical protein

Type	Coordinates	Feature
	c(4076606..4081888)	Flagellar hook-length control protein FliK
	c(4082171..4082485)	hypothetical protein
	c(4082624..4082821)	hypothetical protein
	c(4083645..4083938)	hypothetical protein
	c(4085228..4085539)	hypothetical protein
	c(4087348..4089435)	hypothetical protein
	c(4089607..4090350)	Ribonucleotide reductase of class II (coenzyme B12-dependent), alpha subunit (EC 1.17.4.1)
	c(4090603..4091193)	Histone protein
	c(4091308..4093452)	Ribonucleotide reductase of class II (coenzyme B12-dependent) (EC 1.17.4.1)
	c(4093692..4093865)	hypothetical protein
	4093884..4095257	HtrA protease/chaperone protein / Serine protease (Protease DO) (EC 3.4.21.-)
	c(4095239..4095361)	hypothetical protein
	4095592..4096809	Phosphate ABC transporter, periplasmic phosphate-binding protein PstS (TC 3.A.1.7.1)
	4096821..4097522	Putative FMN hydrolase (EC 3.1.3.-); 5-Amino-6-(5'-phosphoribitylamino)uracil phosphatase
	4097868..4099244	Peptidase B (EC 3.4.11.23)
	c(4100433..4101035)	Probable transmembrane protein
	4101184..4102920	Peptidase M1 family protein
Genome island 13 (4379500..4407599)	4379108..4381906	Serine/threonine protein kinase
	4382155..4382568	hypothetical protein
	c(4382682..4382993)	hypothetical protein
	c(4383009..4383341)	hypothetical protein
	c(4385005..4386333)	McrBC 5-methylcytosine restriction system component
	c(4386348..4388516)	conserved domain protein
	c(4388731..4391874)	Type I restriction-modification system, restriction subunit R (EC 3.1.21.3)
	c(4393736..4396318)	Type I restriction-modification system, DNA-methyltransferase subunit M (EC 2.1.1.72)

Type	Coordinates	Feature
	c(4396638..4396713)	tRNA-Met-CAT
	4396882..4397238	Protein of unknown function DUF1428
	c(4397437..4397655)	hypothetical protein
	4397935..4398078	hypothetical protein
	c(4398279..4398428)	hypothetical protein
	4398303..4399745	hypothetical protein
	4399921..4400949	hypothetical protein
	4401154..4401525	hypothetical protein
	c(4401637..4406280)	Lhr-like helicases
	4406485..4408746	TonB-dependent receptor
