# Supplementary material for: The genome sequence of Dyella jiangningensis FCAV SCS01 from a lignocellulose-decomposing microbial consortium metagenome reveals potential for biotechnological applications
Source: Genet Mol Biol. 2018 May 14;41(2):507–13. doi: 10.1590/1678-4685-GMB-2017-0155 (PMC6082245; doi:10.1590/1678-4685-GMB-2017-0155)
Supplement: Supplementary file 5 [file 1415-4757-GMB-10159016784685GMB20170155-s005.pdf]

Supplementary Material to "The genome sequence of *Dyella jiangningensis* FCAV SCS01 from a lignocellulose-decomposing microbial consortium metagenome reveals potential for biotechnological applications"

Table S5. The 371 exclusive genes found in *Dyella jiangningensis* FCAV SCS01. Genes showing potential for lignocellulose decomposition are in bold

**Putative product**	**N#**
Hypothetical proteins	265
5'-nucleotidase YjjG (EC 3.1.3.5)	1
ABC-type Fe3+ transport system protein; Molybdenum transport protein	2
Alcohol dehydrogenase (EC 1.1.1.1)	1
**Alpha-glucosidase (EC 3.2.1.20)**	1
Beta-lactamase (EC 3.5.2.6)	1
Chemotaxis response regulator containing a CheY-like receiver domain and a methylesterase domain	1
Chemotaxis protein methyltransferase CheR (EC 2.1.1.80)	1
Chitosanase precursor (EC 3.2.1.132)	1
Cobalt-zinc-cadmium resistance protein CzcA; Cation efflux system protein CusA	2
Cobalt-zinc-cadmium resistance protein CzcD	1
Conjugative transfer protein TrbB	1
Conjugative transfer protein TrbC	1
Conjugative transfer protein TrbD	1
Conjugative transfer protein TrbE	1
Conjugative transfer protein TrbF	1
Conjugative transfer protein TrbG	1
Conjugative transfer protein TrbI	1
Conjugative transfer protein TrbJ	1
Conjugative transfer protein TrbL	2
Cyclic beta-1,2-glucan modification transmembrane protein	1
Cytochrome c oxidase polypeptide I (EC 1.9.3.1)	1
Cytochrome c oxidase polypeptide II (EC 1.9.3.1)	1

**Putative product**	**N#**
Cytochrome c oxidase polypeptide III (EC 1.9.3.1)	1
Cytochrome c oxidase polypeptide IV(EC 1.9.3.1)	1
Cytochrome c-type biogenesis protein DsbD, protein-disulfide reductase (EC 1.8.1.8)	1
Cytosine deaminase (EC 3.5.4.1)	1
DNA primase (EC 2.7.7.-), phage-associated	1
DNA-invertase	1
D amino acid oxidase (DAO) family (EC 1.4.3.3)	1
D-alanyl-D-alanine carboxypeptidase (EC 3.4.16.4)	1
Membrane protein	3
Ferrichrome-iron receptor	1
Flagellar hook-length control protein FliK	2
Fumarylacetoacetate hydrolase family protein	1
GerE family regulatory protein	1
Glutamine synthetase adenylyltransferase	1
Glycosyl transferase, group 2 family protein	1
Glyoxalase family protein	1
IS, phage, Tn; Transposon-related functions	1
Integrase	1
**Maltodextrin glucosidase (EC 3.2.1.20)**	1
McrBC 5-methylcytosine restriction system component	1
Methyltransferase type 12	1
Methyl-accepting chemotaxis protein	1
Mobile element protein	2
Molybdopterin oxidoreductase subunit, predicted; chaperone protein HtpG	1
Molybdopterin oxidoreductase (EC 1.2.7.-)	1
Molybdopterin oxidoreductase, iron-sulfur binding subunit (EC 1.2.7.-)	1
Muconate cycloisomerase (EC 5.5.1.1)	1
Nicotinamidase family protein YcaC	1
N-acyl-D-amino-acid deacylase(EC:3.5.1.81)	1

**Putative product**	**N#**
Outer membrane protein	1
O-antigen ligase	1
PQQ-dependent oxidoreductase, gdhB family	1
Probable transmembrane protein	1
Phytanoyl-CoA dioxygenase	1
Pole remodelling regulatory diguanylate cyclase	1
**Predicted maltose transporter MalT**	1
**Predicted maltose-specific TonB-dependent receptor**	1
Probable cytochrome c2	1
Protein containing plastocyanin/azurin family domain	1
Quaternary ammonium compound-resistance protein SugE	1
RND efflux system, outer membrane lipoprotein CmeC	1
Rossmann fold nucleotide-binding protein Smf possibly involved in DNA uptake	1
SCO1/SenC family protein	1
TPR repeat	1
TonB-dependent receptor	1
Transcriptional regulator	2
Transcriptional regulator containing an amidase domain and an AraC-type DNA-binding HTH domain	1
**Transcriptional regulator of maltose utilization, LacI family**	1
Transcriptional regulator, AraC family	2
Transcriptional regulator, ArsR family	1
Transcriptional regulator, LysR family	1
Transcriptional regulator, RpiR family	1
Two-component system response regulator	1
**UDP-glucose dehydrogenase (EC 1.1.1.22)**	1
VgrG protein	1
autotransporter	3
diguanylate cyclase/phosphodiesterase (GGDEF & EAL domains) with PAS/PAC sensor(s)	1
dolichol-phosphate mannosyltransferase	1

**Putative product**	**N#**
extensin-like protein	1
**Glycosyltransferase (EC:2.4.-)**	1
**hydrolase, CocE/NonD family**	1
methyltransferase	1
polysaccharide biosynthesis protein	1
probable alpha helix chain yaiN	1
probable disulphide-isomerase	1
probable integral membrane protein NMA0260	1
probable two-component response regulator	2
putative lipoprotein	1
sensory box histidine kinase/response regulator	1
Surface protein	1
tributyrin esterase	1
virulence-associated protein E	1
